# Neural Correlates of Physical Activity Moderate the Association Between Problematic Mobile Phone Use and Psychological Symptoms

**DOI:** 10.3389/fnbeh.2021.749194

**Published:** 2022-02-01

**Authors:** Liwei Zou, Xiaoyan Wu, Shuman Tao, Yajuan Yang, Qingjun Zhang, Xuedong Hong, Yang Xie, Tingting Li, Suisheng Zheng, Fangbiao Tao

**Affiliations:** ^1^Department of Radiology, The Second Hospital of Anhui Medical University, Hefei, China; ^2^Department of Maternal, Child and Adolescent Health, School of Public Health, Anhui Medical University, Hefei, China; ^3^MOE Key Laboratory of Population Health Across Life Cycle, Hefei, China; ^4^NHC Key Laboratory of Study on Abnormal Gametes and Reproductive Tract, Hefei, China; ^5^Anhui Provincial Key Laboratory of Population Health and Aristogenics, Hefei, China; ^6^Department of Nephrology, The Second Hospital of Anhui Medical University, Hefei, China; ^7^School of Nursing, Anhui Medical University, Hefei, China; ^8^Ping An Healthcare Diagnostics Center, Hefei, China

**Keywords:** MRI, moderating analysis, physical activity, depression, smartphone addiction

## Abstract

**Background:**

Despite the evidence of an association between problematic mobile phone use (PMPU) and psychological symptoms, a few studies explore whether physical activity (PA) could moderate the effect of PMPU on psychological symptoms and its neural substrates. The aim of this study was to examine the association between PMPU and psychological symptoms in late adolescents, along with the potential moderating effect of PA and neural basis by brain gray matter volume (GMV).

**Methods:**

A total of 251 college students reported on their PMPU, PA, and psychological symptoms and subsequently underwent structural magnetic resonance imaging to explore the neural basis of their PA characteristics. A multiple regression model was performed to detect brain GMV associated with PA by the voxel-based morphometry (VBM) method. Moderating analysis was conducted using PROCESS macro in the SPSS software.

**Results:**

Behavioral results showed that PMPU was correlated to depression, anxiety, and stress, and PA has significantly moderated the association between PMPU with depression, anxiety, and stress. The VBM analysis showed that PA was correlated to GMV of the right fusiform gyrus (FFG), left precuneus (PCUN), left insula (INS), and left triangular part of inferior frontal gyrus (IFGtriang). Moreover, GMV of the left INS moderated the relationship between PMPU and depression.

**Conclusion:**

This study has shed light on the neural perspective of PA that moderates the relationship between PMPU and depressive symptom.

## Introduction

Currently, the young generation is experiencing rapid developments in emerging technologies, especially portable electronic devices, i.e., mobile phones. The mobile phone has become an indispensable part of our daily life due to accessing the Internet. It is reported that the prevalence of mobile phone use is 90.8% globally from GlobalWebIndex^1^. In China, there were 985.8 million mobile phone users by the end of December in 2020; 21.0% of whom were students (CNNIC, 2021). Thus, the mobile phone use is attracting a growing concern and is drawing research attention.

Along with many advantages of mobile phones with access to information and fast communication, many studies discussed the potential negative consequences of mobile phone overuse in recent years. Problematic mobile phone use (PMPU) has also been as termed mobile phone dependence and mobile phone addiction (Derevensky et al., 2019), which is defined as excessive use with features of craving, tolerance, and dependence that resulted in adverse health and functional consequences (Billieux et al., 2015; De-Sola Gutierrez et al., 2016; Elhai et al., 2019).

Recent evidence indicates that PMPU has been associated with bodily pain, sleep problems, and mental health (Demirci et al., 2015; Lissak, 2018; Ng et al., 2020). Multiple studies have documented a significant association between PMPU and mental disorders in adolescents. For example, a longitudinal study by Lapierre et al. (2019) found the association between PMPU and depression in late adolescents. Similarly, the studies on Korean, Serbia, Italian, and American adolescents reported that the intensity of mobile phone use was significantly associated with anxiety and depression (Visnjic et al., 2018; Grant et al., 2019; Park et al., 2019). Moreover, a systematic review included ten studies that found the most support for relationships between PMPU and depression and anxiety severity in different samples of adolescents and adults (Elhai et al., 2017).

The PMPU and physical inactivity were common health risk behaviors. The preceding study found that PMPU might directly or indirectly disrupt physical activity (PA) among college students (Lepp et al., 2013). Moreover, several studies also showed an obvious reverse association between PA and PMPU (Kim et al., 2015; Penglee et al., 2019). Another study found that PMPU increased the likelihood of sedentary behavior and reduced exercise intensity during PA (Jacob and Barkley, 2016).

At present, it has attracted more attention to PA as a treatment for mental health disorders (Zschucke et al., 2013). In a large cross-sectional study (Chekroud et al., 2018), individuals who exercised had better mental health than individuals who did not exercise. Moreover, individuals who were engaged on regular leisure-time exercise developed less depression from a large cohort study (Harvey et al., 2018). In addition, meta-analysis from randomized clinical trials had reported that PA is associated with reduced depressive symptom and supported that PA is a treatment for depression (Schuch et al., 2016).

Although such studies indicated that PA could reduce psychological symptoms, the mechanism of protective role of PA for mental health disorders was still unclear. There is a growing evidence of using functional magnetic resonance imaging (fMRI) technology to detect the neural basis of PA. Several recent studies have found the relationship between PA and brain morphology (Thomas et al., 2016; Rosano et al., 2017). We, therefore, hypothesized that a relationship between PMPU and psychological symptoms is moderated by gray matter volume (GMV) of PA-related brain regions.

The aim of this study was (a) to examine whether PA would moderate the relationship between PMPU and psychological symptoms and (b) to examine the neural correlates of PA and whether these neural correlates would moderate the relationship between PMPU and psychological symptoms as well.

## Materials and Methods

### Participants

**FIGURE 1:**
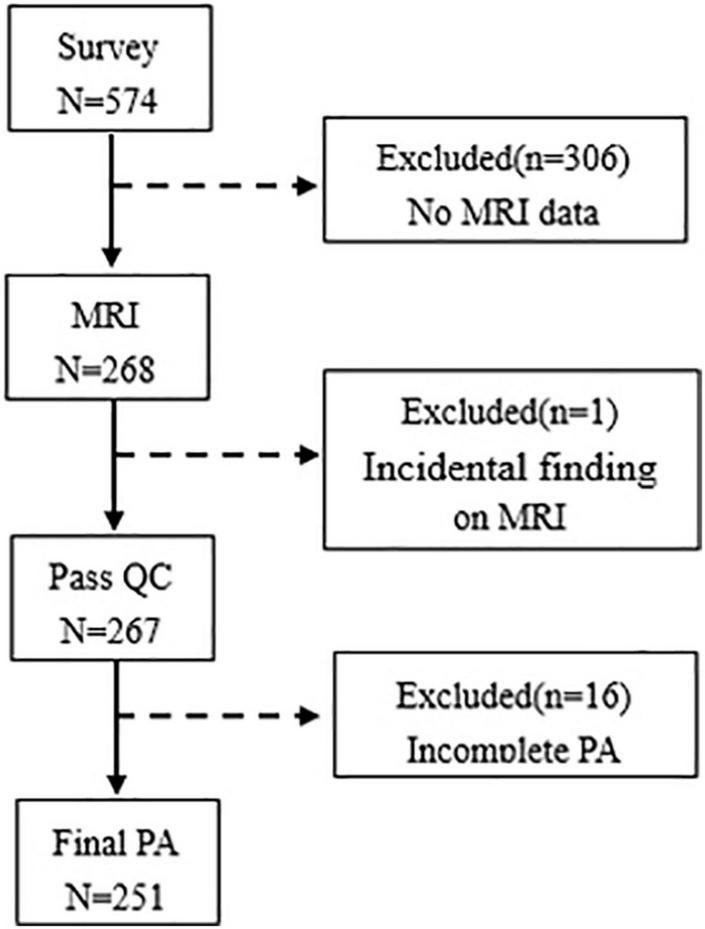
Flowchart of data exclusion is given in this study. QC, quality control; PA, physical activity.

This cross-sectional survey was performed among freshmen from 2 schools and 5 different majors at one university in Hefei, Anhui Province, from April 2019 to June 2019. Data were collected from 574 participants, and 268 participants were obtained the MRI scan in this study. Of the 268 college students, a total of 17 were excluded due to incidental finding (*n* = 1) and missing information on PA (*n* = 16), and the sample of this study included 251 college students (mean age: 19.01 ± 0.85 years, 20.72% men). A flowchart shows the exclusion of data in Figure 1. This study was approved by the Ethics Committee of Anhui Medical University, and all participants provided written informed consent.

### Measurement

#### Assessment of Problematic Mobile Phone Use

The Self-Rating Questionnaire for Adolescent PMPU (SQAPMPU) (Tao et al., 2013) was a 13-item measure that included three dimensions, namely, withdrawal symptoms, craving, and physical and mental health status. Each item was rated on a 5-point Likert-type scale (not true at all = 1, slightly true = 2, moderately true = 3, strongly true = 4, and extremely true = 5), so that the total score possibly ranged from 13 to 65. Higher scores indicated higher levels of PMPU and defined PMPU with SQAPMPU score of ≥ 29 (75th percentile) in this study. The Cronbach’s alpha coefficient of the scale was 0.89.

#### Assessment of Physical Activity

The PA was assessed by the 7-item Chinese version of the International PA Questionnaire (IPAQ-C) (Macfarlane et al., 2007), which was classified into three types of PA, namely, walking, moderate PA (MPA; e.g., carrying a light load, swimming, and cycling), and vigorous PA (VPA; e.g., carrying or lifting heavy loads, digging, and running). Participants were obtained the frequency (days per week) and duration (minutes per day) of each activity during the last 7 days. The amount of PA was processed into metabolic equivalent (MET) min/week using MET values for walking (3.3), MPA (4.0), and VPA (8.0). The MET min/week of each activity was calculated by its value of MET × frequency (day/week) × duration (min/day). The total PA was a sum of total (walking + MPA + VPA) MET min/week.

#### Assessment of Psychological Symptoms

The Chinese version of the Depression Anxiety Stress Scale-21 (DASS-21) assessed the psychological symptoms in college students. The DASS-21 consisted of 21 items for measuring depressive, anxiety, and stress symptoms. Each subscale was measured by 7 items with 4 response options from 0 (not at all) to 3 (very much or most of the time). The total score possibly ranged from 0 to 63, and higher scores indicated severe psychological symptoms. The depressive, anxiety, and stress symptoms were defined by 9, 7, and 14 scores in each subscale (Wang K. et al., 2016). The Cronbach’s alpha coefficient values of this study were 0.85 for depression, 0.78 for anxiety, and 0.84 for stress.

### Magnetic Resonance Imaging

#### Image Acquisition

All MRI data were acquired using a 3.0 T Philips Ingenia CX scanner (Philips, Best, Netherlands) in the Ping An Healthcare Diagnostics Center (Hefei, Anhui, China). Polyurethane foam pads and earmuffs were used to minimize head motion and reduce scanner noise during scanning. The three-dimensional (3D) high-resolution T1-weighted structural images were acquired using the fast field echo (FFE) technique by the following parameters: echo time = 3.2 ms, repetition time = 7.1 ms, field of view = 256 mm × 256 mm, slice thickness = 1 mm, voxel size 1 mm × 1 mm × 1 mm, and the number of slices = 180. The acquisition time was 5 min and 5 s.

#### Data Preprocessing

Voxel-based morphometry (VBM) of 3D high-resolution T1-weighted structural images was performed using the Computational Anatomy Toolbox (CAT^2^) and Statistical Parametric Mapping (SPM12^3^). We viewed each T1-weighted image in SPM to screen for gross anatomical abnormalities or head motion artifacts. The main process of VBM included the segmentation of structural images into GM, white matter (WM), and cerebrospinal fluid (CSF), normalization by diffeomorphic anatomical registration using exponentiated lie algebra (DARTEL) method, and smoothing of GMV segments with a 6 mm full width at half maximum (FWHM) isotropic Gaussian kernel.

### Statistical Analysis

Data analysis was conducted using SPSS version 23.0 (SPSS, Chicago, IL, United States). The descriptive statistics used were mean (SD) and median for continuous variables and frequencies and percentages for categorical variables. We tested the normality of the regression standardized residual when taken regression analyze. The statistical significance was set at *p* < 0.05.

To address our hypothesis, we conducted the following analysis steps.

First, linear regression analysis was conducted to explore the association between PMPU and psychological symptoms, including depressive, anxiety, and stress scores as outcomes, and SQAPMPU scores as predictors (step 1).

Second, we examined whether PA moderates the association between PMPU and psychological symptoms. Prior to moderation analyses, PA from total MET was log-10 transformed; PMPU and PA were mean-centered to reduce multicollinearity (Aiken et al., 1991). A moderation analysis was conducted with PMPU as an independent variable, PA as a moderator, and psychological symptoms as a dependent variable using SPSS PROCESS macro, version 3.0 (model 1), developed by Hayes (step 2) (Sanchez-Martinez and Otero, 2009).

Third, we used whole-brain voxel-based multiple regression analyses (based on general linear model) using SPM12 software with voxel-wise GMV value as a dependent variable and PA as a covariate of interest to investigate the brain regions where the GMV was related to PA. Moreover, age, gender, and total intracranial volume were included as covariates of no interest (step 3). The results were set the significant value at *p* < 0.05 (height threshold of *p* < 0.001) with family-wise error (FWE) correction for multiple comparisons.

Finally, we aimed to assess whether PA-related GMV moderates the association between PMPU and psychological symptoms. Therefore, we extracted GMV from brain regions that were strongly (*p* < 0.05, FWE-corrected) associated with PA. The procedure was the same as above, except for the extracted GMV that correlated to PA as a moderator. Simple slopes were used to show the association between PMPU and psychological symptoms at low (*M* − 1 *SD*) and high (*M* + 1 *SD*) levels of the moderator.

## Results

In this study, 13.1, 23.1, and 7.2% of participants had depressive, anxiety, and stress symptoms, respectively. Table 1 presents the demographic characteristics of different psychological symptoms.

**TABLE 1:** Demographic characteristics of the participants.

Variables	*N*	Depression symptoms			Anxiety symptoms			Stress symptoms		
		%	χ*^2^*	*P*	%	χ*^2^*	*P*	%	χ*^2^/Fishe’s test*	*P*
Gender			0.01	0.940		0.14	0.707		–	0.771
Male	52	13.5			21.2			7.7		
Female	199	13.1			23.6			7.0		
Residential area			0.16	0.687		1.13	0.288		1.41	0.235
Rural	145	12.4			20.1			5.5		
Urban	106	14.2			26.4			9.4		
Any siblings			0.01	0.917		0.22	0.640		0.11	0.742
Yes	55	12.7			25.5			9.1		
No	196	13.3			22.4			6.6		
Perceived family income			4.66	0.098		10.22	0.006		0.45	0.905
Low	49	16.3			30.6			8.2		
Medium	189	11.1			19.0			6.9		
High	13	30.8			53.8			7.7		
Academic performance			0.72	0.699		0.03	0.985		3.79	0.134
Poor	45	11.1			22.2			13.3		
Medium	158	12.7			23.4			5.1		
Good	48	16.7			22.9			8.3		
Father’s educational level			4.61	0.100		1.93	0.381		0.30	0.928
Primary school or lower	49	22.4			30.6			8.2		
Middle school	174	10.9			21.3			6.9		
College or above	28	10.7			21.4			7.1		
Mother’s educational level			0.10	0.953		0.21	0.899		0.07	0.966
Primary school or lower	119	12.6			21.8			6.7		
Middle school	119	13.4			24.4			7.6		
College or above	13	15.4			23.1			7.7		

### Association of Problematic Mobile Phone Use With Psychological Symptoms

Linear regression analyses showed a significant relationship between PMPU with depressive [β = 0.32, *F*(1,249) = 72.19, *p* < 0.001, *R*^2^ = 0.23], anxiety [β = 0.35, *F*(1,249) = 89.66, *p* < 0.001, *R*^2^ = 0.27], and stress symptoms [β = 0.37, *F*(1,249) = 73.26, *p* < 0.001, *R*^2^ = 0.23].

### Association of Physical Activity With Gray Matter Volume

**FIGURE 2:**
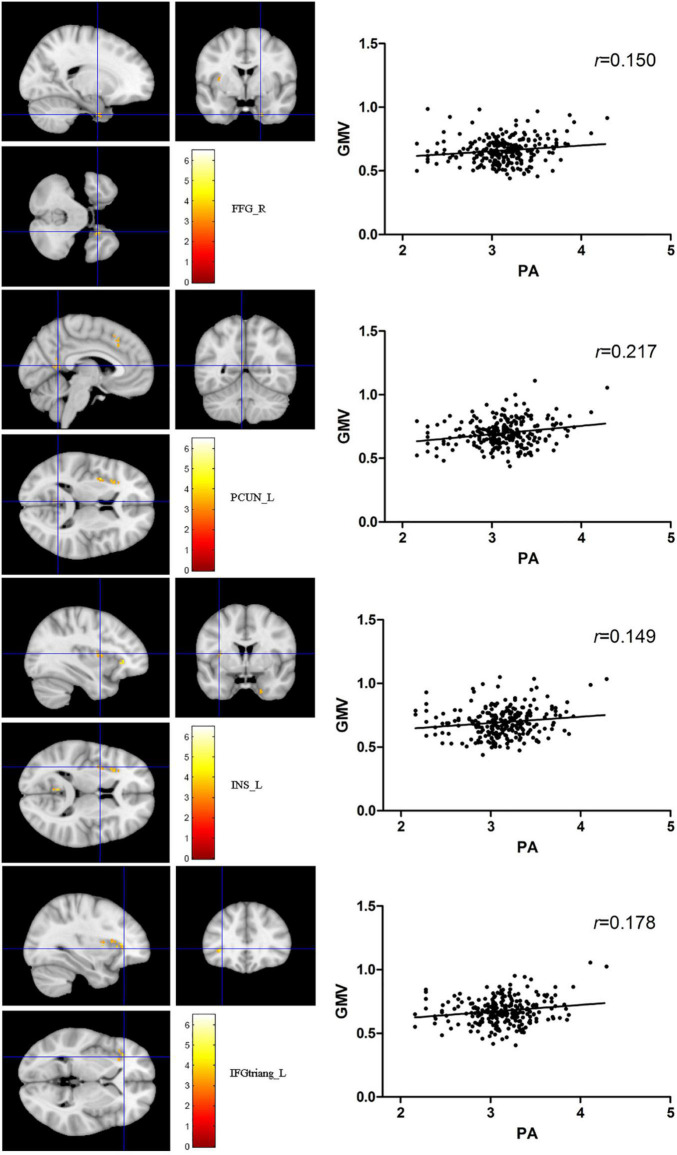
The red-yellow color indicates positive association between total PA MET and gray matter volume (GMV) in the right fusiform gyrus, left precuneus, the left insula (INS), and left triangular part of inferior frontal gyrus. The color scale represents *t* values. The threshold for displaying was set to *p* < 0.05, family-wise error corrected.

The total PA was positively correlated to several brain regions, including the right fusiform gyrus (FFG), the left precuneus (PCUN), the left insula (INS), and the left triangular part of inferior frontal gyrus (IFGtriang) (*P*_FWE_ < 0.05 for all results; Table 2 and Figure 2). No negative association results were observed.

**TABLE 2:** Brain regions in which GMV was significantly correlated to total PA.

Cluster	Region	Cluster size	Peak MNI (mm)			Peak *T* value
			*X*	*Y*	*Z*	
1	FFG_R	40	19.5	−1.5	−37.5	4.30
2	PCUN_L	43	−4.5	−54	10.5	4.09
3	INS_L	47	−34.5	1.5	10.5	4.08
4	IFGtriang _L	135	−31.5	31.5	1.5	5.53

*Height threshold p < 0.001, corrected for FWE, cluster size = 40. FFG, fusiform gyrus; PCUN, precuneus; INS, insula; IFGtriang, inferior frontal gyrus, triangular part; R, right; L, left; FWE, family-wise error.*

### Association of Problematic Mobile Phone Use With Psychological Symptoms Is Moderated by Physical Activity and Physical Activity-Related Gray Matter Volume

**FIGURE 3:**
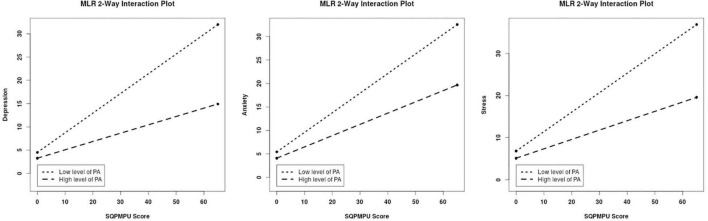
PA moderated the association between problematic mobile phone use (PMPU) and psychological symptoms. Simple slopes are plotted at low (M − 1 SD) and high (M + 1 SD) PA.

**FIGURE 4:**
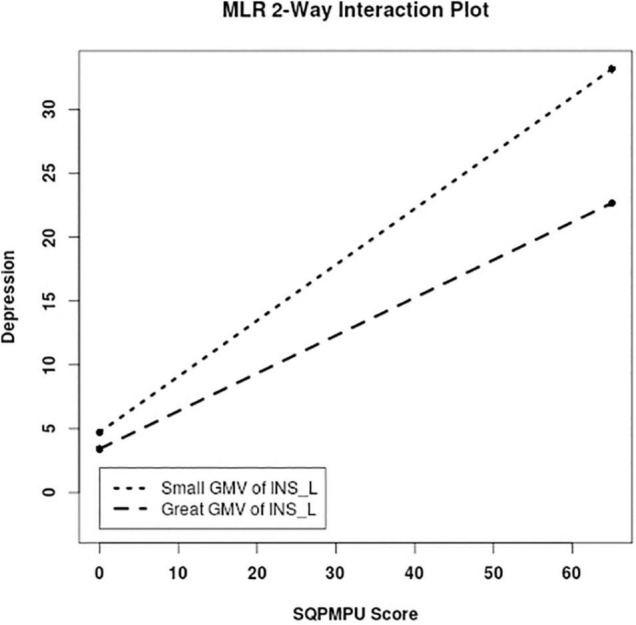
PA-related GMV moderated the association between PMPU and psychological symptoms. Simple slopes are plotted at low (M − 1 SD) and high (M + 1 SD) GMV of the left INS that correlated to PA level.

For moderation analysis, we tested whether the association between PMPU and psychological symptoms was reduced by introducing PA and GMV of PA-related brain regions. As a result, we found the significantly moderate effect of PA on the association between PMPU with depressive (Δ*R*^2^ = 0.030, *F* = 10.096, *p* = 0.002), anxiety (Δ*R*^2^ = 0.016, *F* = 5.796, *p* = 0.017), and stress (Δ*R*^2^ = 0.024, *F* = 8.160, *p* = 0.005) symptoms (Table 3 and Figure 3). In contrast, for GMV of PA-related brain regions, we found that the left INS (Δ*R*^2^ = 0.013, *F* = 4.145, *p* = 0.043) had significantly moderated the association between PMPU and depressive symptom (Table 3 and Figure 4) and did not find the moderate effect of any other PA-related brain regions on anxiety or stress symptom.

**TABLE 3:** Results from the moderated regression analysis predicting psychological symptoms by adjusted age, gender, residential area, any siblings, perceived family income, academic performance, and educational level of parents.

	Predictors	Depression				Anxiety				Stress			
		β	*t*	ΔR^2^	*F*	β	*T*	ΔR^2^	*F*	β	*t*	ΔR^2^	*F*
1	PMPU	0.300	7.906**	0.030	10.096**	0.334	9.179**	0.016	5.796*	0.347	8.074**	0.024	8.160**
	PA	–1.709	–1.935			–1.657	–1.956			–2.446	−2.446*		
	PMPU × PA	–0.355	−3.177*			–0.258	−2.407*			–0.361	−2.857**		
2	PMPU	0.323	8.421**	0.005	1.524	0.354	9.530**	0.000	0.055	0.380	8.661**	0.001	0.417
	FFG_R	4.861	1.591			3.209	1.098			6.891	1.994*		
	PMPU × FFG_R	0.468	1.234			0.085	0.235			0.277	0.646		
3	PMPU	0.316	8.222**	0.004	1.286	0.347	9.449**	0.001	0.265	0.367	8.396**	0.001	0.450
	PCUN_L	2.733	0.922			–0.611	–0.216			2.092	0.621		
	PMPU × PCUN_L	0.417	1.134			0.181	0.515			0.280	0.671		
4	PMPU	0.323	8.490**	0.013	4.145*	0.353	9.671**	0.005	1.777	0.371	8.473**	0.002	0.546
	INS_L	–6.258	−2.288*			4.744	1.809			3.653	1.163		
	PMPU × INS_L	–0.672	−2.036*			0.422	1.333			0.280	0.739		
5	PMPU	0.316	8.115**	0.001	0.346	0.346	9.356**	0.004	1.458	0.368	8.348**	0.001	0.211
	IFGtriang_L	2.127	0.691			1.890	0.647			3.074	0.882		
	PMPU × IFGtriang_L	–0.248	–0.588			–0.482	–1.207			–0.219	–0.459		

*p < 0.05, **p < 0.01.

## Discussion

To the best of our knowledge, this is the first study to detect the moderate effect of PA and PA-related neural structure on the relationship between PMPU and psychological symptoms in college students. The results had indicated that increasing PA could reduce the association between PMPU and psychological symptoms, and greater GMV of the left INS which correlated to PA could also reduce the relationship between PMPU and depressive symptom. The results support our hypothesis that the relationship between PMPU and psychological symptoms could be moderated by PA, and GMV of which is correlated to PA in college students.

Several studies focused the role of psychological factors on the relationship between PMPU and psychological symptoms. For example, Elhai et al. (2020) recruited 1,034 undergraduate students, used a structural equation model, and demonstrated that fear of missing out significantly mediated the relationship between problematic smartphone use and anxiety. Our previous study (Tao et al., 2017; Zou et al., 2019) found that poor sleep quality may increase the risk of psychological symptoms in students with PMPU than non-PMPU students.

Growing evidence suggests the involvement of brain structural abnormality in PMPU and mental disorders. For PMPU, a recent study by Horvath et al. (2020) has found that smartphone addiction group had lower GMV in the left anterior INS, inferior temporal, and parahippocampal cortex than healthy controls. Moreover, this study reports smaller GMV in the right lateral orbitofrontal cortex in problematic smartphone users than controls (Lee et al., 2019). Another study also has used the VBM method and found decreased GMV in the right superior frontal gyrus, the right inferior frontal gyrus, and bilateral thalamus in the mobile phone dependence group (Wang Y. et al., 2016). For mental disorders, a meta-analysis that included 41 studies has reported GMV difference in major depression compared with healthy controls including INS and anterior superior temporal gyrus (Wise et al., 2017). Another meta-analysis conducted structural findings across multiple mental disorders, including schizophrenia, bipolar disorder, depression, addiction, obsessive-compulsive disorder, and anxiety, and it identified a concordance of GMV loss across the anterior INS and dorsal anterior cingulate cortex (Goodkind et al., 2015). Therefore, disruption in neural structure may contribute to the relationship between PMPU and psychological symptoms.

It is well documented that PA has a key role in the recovery of stroke patients of movement through brain plasticity (Hara, 2015). For healthy participants, several cross-sectional studies have also indicated that exercises are associated with greater volume in hippocampus (Firth et al., 2018) and anterior cingulate cortex (Bento-Torres et al., 2019). It has also suggested that exercise interventions can increase the volume of prefrontal and anterior cingulate cortex from randomized controlled trials (Ruscheweyh et al., 2011). In addition, recent systematic reviews have shown that exercise can increase the volumes in hippocampus and several cortical regions (Firth et al., 2018; Zheng et al., 2019). Again, the available data have supported that self-reported PA is positively related to GMV and, most consistently, to frontal cortex and medial temporal lobe, so do our results, i.e., FFG, PCUN, INS, and IFGtriang.

Nevertheless, the studies are limited to detect the moderating effect of PA and PA-related neural structure on the relationship between PMPU and psychological symptoms in college students. Our findings show that a high level of PA can reduce the relationship between PMPU and psychological symptoms, and greater GMV of the left INS that correlated to PA has the same protective role in the relationship between PMPU and depressive symptom. In the context of the literature, this study expands the evidence of some neuroplastic mechanisms on PA that moderated the relationship between PMPU and psychological symptoms.

The INS cortex is well known for an important part of “salience” network and functions involved in interoception, autonomic control, emotional guidance of social behavior, and perceptual self-awareness (Benarroch, 2019). It has been supported that INS cortex is strongly correlated to addictive behaviors, because addictive behaviors are involved in the decision-making process such as choosing immediate rewards that are always associated with physiological states that cause strong interoceptive signals (Droutman et al., 2015). In contrast, comparative quantitative meta-analysis had been performed to find a common core of areas, i.e., dorsal anterior cingulate cortex and INS are affected across most mental disorders (Downar et al., 2016). Thus, the INS maybe a key node that links the addictive behaviors and mental health.

Key strengths of this study are neuroimaging-based and relatively large sample size, along with the relatively complete information collected and the power to control multiple confounding factors. However, several limitations should be acknowledged. First, this is a cross-sectional design study, limiting inferences on directionality to any of the associated factors, although we assumed that the directionality is from behavior to psychological symptoms. Second, self-reported measurements will lead to recall bias, and more objective measures should be taken in the future, such as accelerometer for PA. Finally, it also remains some possible residual confounding factors (e.g., environmental factors), and the results were not substantially changed after controlling some potential confounders.

This study demonstrates that high levels of PA can reduce the association between PMPU and psychological symptoms and further finds that the GMV of the left INS which is correlated to PA may play a key role on the relationship between PMPU and depressive symptom. Future work should take longitudinal design to explore the potential protective factor of PA on the relationship between PMPU and psychological symptoms and detect the neural basis of moderate effects of PA on such a relationship.

## Data Availability Statement

The original contributions presented in the study are included in the article/supplementary material, further inquiries can be directed to the corresponding authors.

## Ethics Statement

The studies involving human participants were reviewed and approved by Ethics Committee of Anhui Medical University. The patients/participants provided their written informed consent to participate in this study.

## Author Contributions

FT designed the study. LZ, XW, ST, YX, TL, and YY performed the survey research. QZ, XH, and SZ conducted MRI and checked the MRI data. LZ, XW, and ST analyzed the data. LZ drafted the manuscript. All authors read and approved the final manuscript.

## Conflict of Interest

The authors declare that the research was conducted in the absence of any commercial or financial relationships that could be construed as a potential conflict of interest.

## Publisher’s Note

All claims expressed in this article are solely those of the authors and do not necessarily represent those of their affiliated organizations, or those of the publisher, the editors and the reviewers. Any product that may be evaluated in this article, or claim that may be made by its manufacturer, is not guaranteed or endorsed by the publisher.

## Footnotes

1
https://www.globalwebindex.com/

2
http://dbm.neuro.uni-jena.de/cat/

3
http://www.fil.ion.ucl.ac.uk/spm/
